# High survivorship rate and good clinical outcomes after high tibial osteotomy in patients with radiological advanced medial knee osteoarthritis: a systematic review

**DOI:** 10.1007/s00402-024-05254-0

**Published:** 2024-03-02

**Authors:** Giacomo Dal Fabbro, Alberto Grassi, Piero Agostinone, Gian Andrea Lucidi, Raschid Fajury, Abhijit Ravindra, Stefano Zaffagnini

**Affiliations:** 1 II Clinica Ortopedica e Traumatologica, IRCCS Istituto Ortopedico Rizzoli, Via G. C. Pupilli 1, Bologna, 40136 Italy; 2 Università di Bologna, Dipartimento di Scienze Biomediche e Neuromotorie DIBINEM, Bologna, Italy

**Keywords:** High tibial osteotomy, HTO, Advanced osteoarthritis, Bone-on-bone, Survivorship, PROMs

## Abstract

**Introduction:**

The role of valgus producing high tibial osteotomy (HTO) for the treatment of advanced knee osteoarthritis (OA) is still controversial. The aim of the current systematic review was to assess survivorship and patient-reported outcomes (PROMs) of high tibial osteotomy in patients with radiological advanced medial knee OA.

**Methods:**

A systematic search of PubMed, Cochrane and EMBASE database was performed in July 2023 in accordance with the Preferred Reporting Items for Systematic reviews and Meta-Analysis (PRISMA) guidelines. Inclusion and exclusion criteria were applied to identify studies investigating the survivorship rate and PROMs of valgus-producing high tibial osteotomy in patients with advanced knee OA at x-ray assessment in the medial compartment at minimum-two-years follow up. Advanced radiological OA was defined as Kellgren Lawrence (K-L) ≥ 3 or Ahlbäch ≥ 2. Survivorship was defined as percentage of patients free of total knee arthroplasty (TKA) at follow-up. Clinical interpretation of provided PROMs were performed according to minimal clinically important difference (MCID) and patient acceptable symptom state (PASS) target values reported in literature. Survivorship data and PROMs scores were extracted, and studies were stratified based on selected study features. The quality of included studies was assessed with modified Coleman score.

**Results:**

A total of 18 studies, totalling 1296 knees with a mean age between 46.9 and 67 years old, were included. Average survivorship was of 74.6% (range 60 − 98.1%) at 10-years follow up. The subjective scoring systems showed good results according to MCID and PASS, and postoperative improvements were partially maintained until final follow-up.

**Conclusion:**

HTO is worth considering as treatment choice even in patients affected by radiological advanced medial knee osteoarthritis. Long term survivorship and good patient reported clinical outcomes could be expected in this population.

**Level of evidence:**

IV; systematic review of level III-IV studies.

## Introduction

Valgus-producing high tibial osteotomy (HTO) represents a wide indication treatment [1] which aims to redistribute weightbearing forces from the medial to the lateral compartment by realignment of the mechanical axis [2]. While it is recognised as an ideal treatment for varus patients with early-to-moderate OA, the role of HTO in advanced OA is still controversial [3, 4]. In fact, while advanced osteoarthritis of the medial compartment had been reported to be a contraindication to performing HTO [5], more recent studies challenged this dogma [6, 7].

While joint replacement is considered the gold standard in treating end-stage OA and represents a successful treatment in older population, it may be less ideal for younger patients. Reported implant survivorship between 5 and 35% at mid- and long-term follow-up and the incidence of complications in younger populations [8, 9], make the indication for joint replacement in younger patients with high expectations more controversial [10]. Furthermore, a strategy of HTO followed by total knee arthroplasty (TKA) showed superior knee survivorship than early TKA at long term in young patients [11]. Unicompartmental knee (UKA) implants, although yielding good medium term outcomes [12], have a rate of revision three time higher than total knee replacement (TKA), very strict indications and do not allow surgeons to fully address malalignment in patients with varus knee [13]. Moreover, since joint replacement is an irreversible metal-oriented solutions, it results in losing any chances of undergoing further conservative or surgical joint treatments after the surgery.

For those reasons, joint preserving treatments should be preferred in mid-aged patients who engage in high demand activities, even in cases of end-stage medial OA. Nevertheless, clear evidence of HTO outcomes in advanced and end-stage OA still lack.

The purpose of this systematic review was to investigate whether HTO is a suitable option for patient with radiologically depicted advanced OA, assessing survivorship and patient reported outcomes (PROMs). The first hypothesis was that HTO in advanced OA would show a high rate of survivorship at mid and long term of follow up. The second hypothesis was that HTO in advanced OA would result in good patient-reported outcomes (PROMs), in particular regarding function and pain scores.

## Methods

### Literature search strategy

The review protocol was recorded in the International Prospective register of Systematic Reviews (PROSPERO, ID: CRD42023440288). Preferred Reporting Items for Systematic Reviews and Meta-Analyses (PRISMA) guidelines were followed in conducting this study [14]. A systematic search on PUBMED, EMBASE, and Cochrane library was performed for the studies investigating the survivorship and patient-reported outcomes of valgus-producing high tibial osteotomy in patients with advanced radiological osteoarthritis (OA) in the medial compartment. Two reviewers (G.D.F. and R.F.) independently conducted the search on 21st August 2023 with the following keywords: (high tibial osteotomy) AND (severe OR advanced OR bone on bone) AND (osteoarthritis). Furthermore, the references of the included studies were examined to verify that all eligible articles were considered. Grey literature was also searched, screening the website clinicaltrials.gov for concluded or ongoing clinical trials related to the topic of the search. The titles and abstracts were also independently screened by the two reviewers (X.X. and X.X.), and the full text of the relevant articles was obtained. Disagreement between reviewer were resolved by a third reviewer (S.Z.).

### Study eligibility

All titles and abstracts were screened with the following inclusion criteria: human clinical studies, opening or closing valgus producing high tibial osteotomy procedure, advanced OA of the medial compartment at x-ray evaluation among included patients (Kellgren-Lawrence ≥ 3, Ahlbäch ≥ 2, or clearly stated advanced OA defined as bone-on-bone or tibiofemoral contact OA), minimum-2-year of follow-up, survivorship and/or patient-reported outcomes analysis, English language, and full text available. In studies encompassing patients with various OA grades which provided a distinguished analysis of the outcomes based on OA degree, only the patient series with advanced radiological OA were included.

Exclusion criteria were as follows: omission of radiological OA grade, non-standard high tibial osteotomy (tibial condylar, Dome or hybrid osteotomy), major concomitant knee surgical procedures (ligaments reconstruction, cartilage replacement procedures), non numerical outcomes scores, and articles that were off topic.

### Data abstraction and synthesis

Extracted data were allocated in a database built in Excel. Each study that met the inclusion criteria was abstracted by two different authors (G.D.F. and A.G.) for the following information: year of publications, study design, number of patients and knees, mean age of included patients, mean follow-up time, osteotomy surgical technique, alignment correction target, radiological evaluation, OA grades included and investigated patient-reported outcomes.

In the included studies in which a distinct analysis was performed for different degree of advanced OA, patient series with different OA degree were abstracted separately. Patients of included studies with a stated medial OA classified as K-L = 4 or Ahlbäch ≥ 3 were classified as “very advanced OA” group. Data regarding the patient reported outcomes were divided based on mean follow up as follows: short term follow up from 24 to 48 months, mid term follow up from 48 to 108 months, and long term follow up from 108 months onwards.

The primary outcome measures were the survivorship and the patient reported outcomes (PROMs) provided at the last follow up. The survival rate was defined as the percentage of HTO that have not been converted to total knee arthroplasty in function of the time. The average of the survivorship percentages at 5- and 10-years follow up weighted in relation to the number of patients was assessed. The clinical interpretation of changes in scores and absolute post-operative values of PROMs was performed according to the definition of minimal clinically important difference (MCID) and patient acceptable symptom state (PASS) provided by literature [15–21].

Due to the low level of evidence of the included studies and to the heterogenous data available it was not possible to perform further quantitative analysis of the abstracted data.

### Risk of bias assessment

**Fig. 1:**
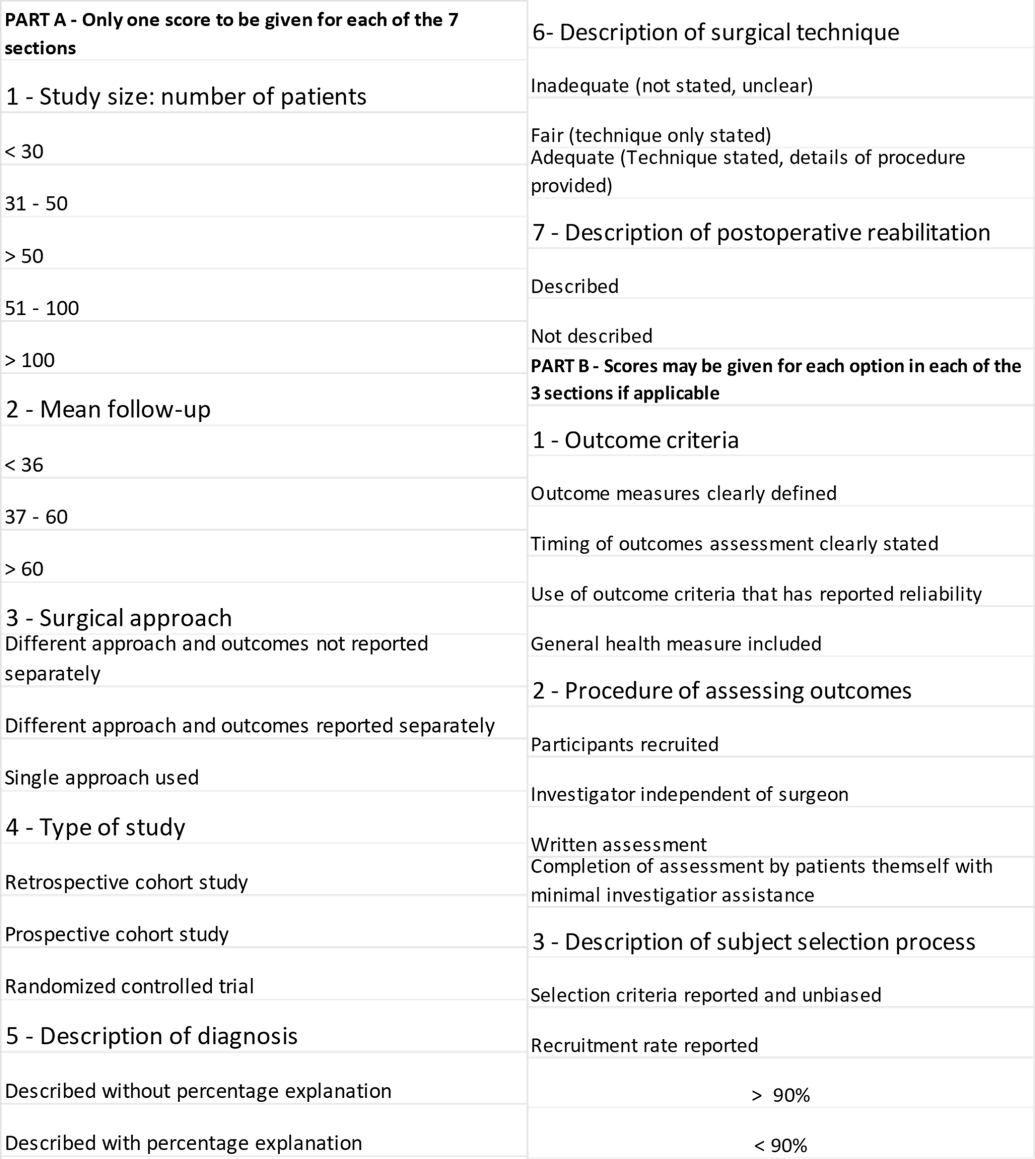
The modified coleman methodology score

The quality of the included studies was assessed using a modified version of the Coleman Methodology Score (CMS) [22, 23], which is composed by 10 criteria, giving a total score between 0 and 100 (Fig. 1). A score of 100 indicates that the assessed study largely avoids chance, various biases and confounding factors. The subsection of the CMS are based on the subsections of the Consolidate Standards of Reporting Trials (CONSORT) statement for randomised controlled trials and are modified to allow to be used for other trial designs. In the current systematic review, the CMS criteria were modified to make them reproducible and relevant for studies about the outcomes of high tibial osteotomy in patients with advanced OA of the knee. Each study was scored by two reviewers (GDF and AR) independently and in duplicate for each of the criteria adopted to give a total CMS between 0 and 100. Disagreements were resolved by discussion.

## Results

### Literature search and included studies

**Fig. 2:**
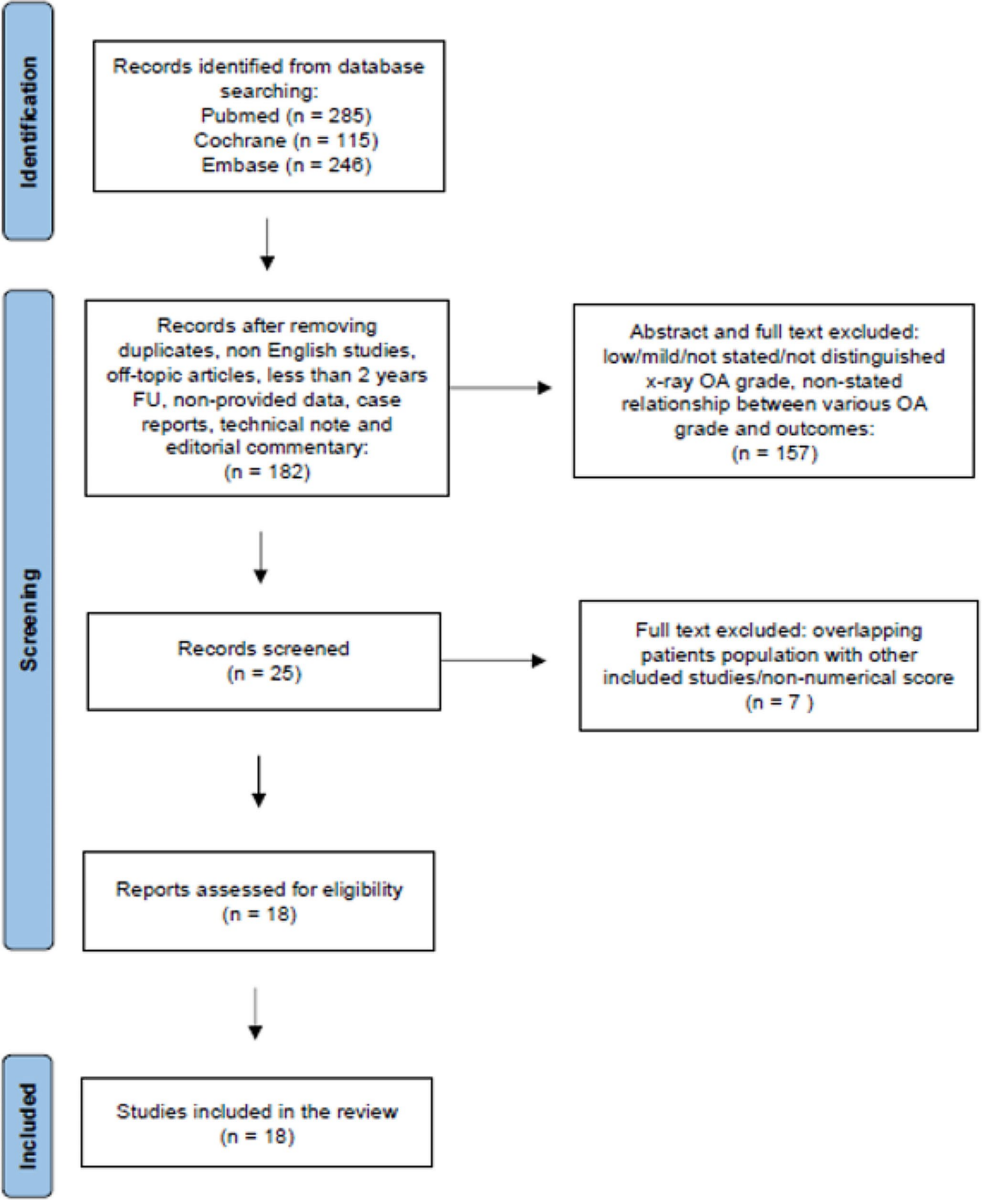
PRISMA flow diagram. n: number of studies; FU: follow up; OA: osteoarthritis

A total of 646 abstracts were identified by the preliminary online research. Duplicates were removed. A total of 464 studies were excluded during the screening of abstract because of: duplicates, non-English articles, off-topic articles, less than two-years-follow-up studies, case reports, technical notes, editorial commentary. A total of 157 articles were not eligible at the full text screening because they did not meet the inclusion criteria, in particular because of: non standard valgus-producing high tibial osteotomy studies (condylar, Dome or hybrid osteotomy), distinguished grade at x-ray among the study population not provided, overlapping patients with other included studies, and non-numerical clinical score (Fig. 2).

A total of 18 studies were included in this systematic review [4, 7, 24–39], including 1296 operated knees with a mean age between 46.9 and 67 years old (Table 1). The Coleman Methodology Score assessment of the included studies is reported in Table 1. The mean follow-up ranged from 24 to 205 months. Eight studies used a knee anterior-posterior weight-bearing radiograph to assess the medial compartment OA, eight studies the full-length double leg standing radiograph, two studies the Rosenberg view radiograph. Eight studies assessed the medial compartment OA with the Kellgren-Lawrence scale, nine with the Ahlbäck scale, and one with the tibiofemoral contact. Among included studies, six series of patients matched the stated criteria of “very advanced OA” [25, 28, 30, 32, 33, 36]. The opening wedge technique was performed in 11 out of 18 studies, a closing wedge technique was performed in 6 studies. In one study both the opening and the closing wedge technique were performed. Seven studies expressed the target correction as the percentage of the medial-to-lateral tibial plateau with respect to weight bearing line with an aimed postoperative range between 50% and 75%, 6 aimed to a mechanical femoral-tibial angle with a range from 0° to 5° of postoperative mechanical valgus, while 4 aimed to an anatomical femoral-tibial angle with a postoperative anatomical valgus between 10° and 12°.

**Table 1:** HTO in advanced OA: included studies

STUDY	STUDY DATA											
	DESIGN	N. OF PATIENTS (M/F)	N. OF KNEES (M/F)	MEAN AGE	MEAN FU (Months)	TECHNIQUE	TARGET CORRECTION	IMAGING	OA GRADING METHOD	OA GRADES INVESTIGATED	OUTCOMES	CMS
Bhan 1992 [24]	Retrospective c.s.	N.R.	35	N.R.	60	CW	3–5° MV	AP WB	Ahlbäck	≥ 2	satisfaction score	81
Cazor 2023-1 [25]	Retrospective c.s.	56	56	56.3 ˜	111,6	OW or CW	0–3° MV	FLDLS	Ahlbäck	2	Survivorship	88
Cazor 2023-2 [25]	Retrospective c.s.	87	87	56.2 ˜	111,6	OW or CW	0–3° MV	FLDLS	Ahlbäck	3	Survivorship	88
Cho 2018 [26]	Retrospective c.s.	17	20 (8/12)	58.4	48.4	OW	62.5% MA	FLDLS	Ahlbäck	≥ 2	KSS, HSS	91
Ekeland 2017 [27]	Prospective Study	N.R.	22	47 ˜	100	OW	62% MA	AP WB	K-L	≥ 3	KOOS	79
Floerkemeier 2013-1 [28]	M. c. retrospective Study	41	41	49˜	43.2	OW	3–5° MV	AP WB	Ahlbäck	≥ 3	OKS	81
Floerkemeier 2013-2 [28]	M. c. retrospective Study	5	5	49˜	43.2	OW	3–5° MV	AP WB	Ahlbäck	2	OKS	81
Hoorntje 2023 [29]	Retrospective c.s.	84	84	55	24	OW	N.R.	AP WB	K-L	≥ 3	OKS	84
Huang 2005-1 [30]	Retrospective c.s.	4	5	57.4 ˜	132	CW	8–10° AV	FLDLS	Ahlbäck	3	Survivorship	80
Huang 2005-2 [30]	Retrospective c.s.	37	41	57.4 ˜	132	CW	8–10° AV	FLDLS	Ahlbäck	2	Survivorship	80
Ishizuka 2021 [31]	Retrospective c.s.	45	56 (11/45)	56.8	205.2	CW	10–12° AV	FLDLS	K-L	≥ 3	Survivorship	88
Kuwashima 2021-1 [32]	Retrospective c.s.	N.R.	47 (42/5)	60.4	122.4	CW	75% MA	AP WB	K-L	4	Survivorship, KSS	80
Kuwashima 2021-2 [32]	Retrospective c.s.	N.R.	81 (63/18)	60.9	120	CW	75% MA	AP WB	K-L	3	Survivorship, KSS	79
Lee 2021 [7]	Retrospective c.s.	44 (14/30)	44 (14/30)	51.6	49.2	OW	57–67% MA	FLDLS	Ahlbäck	≥ 2	KSS, HSS	79
Primeau 2021-1 [33]	Prospective Study	N.R.	134	46.9 ˜	84	OW	50-62.5% MA	FLDLS	K-L	4	Survivorship	91
Primeau 2021-2 [33]	Prospective Study	N.R.	244	46.9 ˜	84	OW	50-62.5% MA	FLDLS	K-L	3	Survivorship	91
Ryu 2018 [35]	Retrospective c.s.	23 (2/21)	23 (2/21)	57.6	40	OW	62.5% MA	Rosemberg	TF contact	Contact between MFC and MTC	VAS, HSS, Lysholm, WOMAC	75
Schuster 2018 [4]	Retrospective c.s.	73	79 (67/12)	50.9	120	OW	0–3° MV	Rosemberg	K-L	≥ 3	Survivorship, IKDC	84
Sohn 2020 [36]	Retrospective c.s.	N.R.	26	56.4 ˜	24	OW	62.5% MA	FLDLS	K-L	4	VAS, WOMAC	73
Takahashi 2002 [37]	Retrospective c.s.	40 (27/13)	55	63.1	123.6	CW	10 ° AV	AP WB	Ahlbäck (Modif.)	≥ 2	HSS	72
Takeuchi 2010 [38]	Prospective Study	24 (6/18)	27	67	61	OW	N.R.	AP WB	Ahlbäck (Modif.)	≥ 2	KSS	70
Van Raaij 2008 [34]	Retrospective c.s.	52	52	49 ˜	144	CW	12° AV	AP WB	Ahlbäck	≥ 2	Survivorship	79
Yoo 2016 [39]	Retrospective c.s.	26 (8/18)	32	49.3	105	OW	3–5° MV	FLDLS	K-L	≥ 3	Survivorship, KSS, Lhysolm	70

HTO: high tibial osteotomy N.: number; FU: follow up; OA: medial osteoarthritis; CMS: Coleman methodology score; M: male; F: female; advanced OA: Kellgren-Lawrence ≥ 3, Ahlbach ≥ 2, or clearly stated advanced OA; -1: patient series 1; -2: patient series 2; c.s.: case series; M.c.: multi-center; OW: opening wedge; CW: closing wedge; AV: anatomical valgus; MV: mechanical valgus; MA: mechanical axis; APWB: anterior-posterior weight bearing knee radiograph; FLDLS: full length double limb standings radiograph; Rosemberg: posterior-anterior weight bearing radiograph at 45° knee bending; K-L: Kellgren-Lawrence; TF: tibio-femoral; MFC: medial femur condyle; MTC: medial tibial condyle; KSS: Knee Society score; HSS: Hospital for Special Surgery score; OKS: oxford knee score; VAS: visual analogue scale for pain; ˜: data obtained from the entire population of the study; N.R.: not reported

### Survivorship

Eight studies provided data about the survivorship (Table 2). The survivorship range was between 96.1% and 99.9% and between 60% and 98.1% at 5- and 10-years follow up, respectively. The weighted average in relations to the number of patients was of 96.3% and 74.6% at 5- and 10-years follow up, respectively.

**Table 2:** Survivorship

STUDY	MEAN AGE	OA	5 YEARS FU	10 YEARS FU	15 YEARS FU	20 YEARS FU
Cazor 2023-2	56.2 ˜	A	N.R.	64%*	N.R.	N.R.
Huang 2005-2	57,4 ˜	A	N.R.	78,1%	75,8%	N.R.
Ishizuka 2021	56.8	A	98.1%	90.1%	83.8%	75.9%
Kuwashima 2021-2	60.9	A	99.9%	98.1%	N.R.	N.R.
Primeau 2021-2	46.9	A	N.R.	74.3%	N.R.	N.R.
Schuster 2018	50.9	A	96.1%	81.7%	N.R.	N.R.
Van Raaij 2008	49 ˜	A	87%	62%	N.R.	N.R.
Yoo 2016	49.3	A	96.9%	N.R.	N.R.	N.R.
Cazor 2023-1	56.3 ˜	VA	N.R	64.2%*	N.R	N.R
Huang 2005-1	57,8 ˜	VA	N.R	60%	50%	N.R.
Kuwashima 2021-1	60.4	VA	97.9%	86.0% ˆ	N.R.	N.R.
Primeau 2021-1	46.9	VA	N.R.	61.9% ˆ	N.R.	N.R.

OA: osteoarthritis; FU: follow-up; A: advanced OA (Kellgren-Lawrence ≥ 3, Ahlbach ≥ 2, or clearly stated advanced OA); VA: very advanced OA (K-L = 4 or Ahlbach ≥ 3); -1: patient series 1; -2: patient series 2; ˆ: significant difference compared with patient series-2 of the same study; ˜: data obtained from the entire population of the study; *: assessed between 12.9 and 14 years of follow-up; N.R.: not reported

### Patient reported outcomes (PROs) (table 3)

**Table 3:** – Patients Reported Outcomes

STUDY	OA	MEAN AGE	MEAN FU (Months)	TECHNIQUE	SCORE	PREOPERATIVE	POSTOPERATIVE
Cho 2018	A	58.4	48.4	OW	KSS knee score KSS function score HSS	70.4 ± 10 58.8 ± 10 70 ± 8.6	95.1 ± 7.6* 81.5 ± 8.8* 90.3 ± 6*
Floerkemeier 2013-1	VA	49˜	43.2	OW	OKS	NR	39.2 ± 7.9
Floerkemeier 2013-2	A	49˜	43.2	OW	OKS	NR	42.8 ± 4.5
Hoorntje 2023	A	55	24	OW	OKS	26.6 ± 8	44 (36–46) *
Kuwashima 2021-1	VA	60.4	122.4	CW	KSS knee score KSS function score	NR NR	NR 55.8 ± 21.2^
Kuwashima 2021-2	A	60.9	120	CW	KSS knee score KSS function score	NR NR	NR 59.5 ± 24.8^
Lee 2021	A	51.6	49.2	OW	KSS knee score KSS function score HSS	NR 63.2 ± 11.6 69.2 ± 9.1	NR 87.8 ± 8.9* 89.4 ± 7.3*
Ryu 2018	A	57.6	40	OW	HSS WOMAC Lysholm VAS	55.6 ± 13.1 57 ± 22.9 50.2 ± 15.7 6.5 ± 1.2	85.1 ± 8.3* 16.5 ± 17.5* 87.4 ± 12* 2.2 ± 1.2*
Sohn 2020	VA	56.4 ˜	24	OW	WOMAC VAS	NR NR	17.4 ± 7.4 3.2 ± 1.4
Takahashi 2002	A	63.1	123.6	CW	HSS	62 ± 12	83 ± 14*
Takeuchi 2010	A	67	61	OW	KSS knee score KSS function score	49 ± 12 58 ± 14	89 ± 7.6* 95 ± 7.6*
Yoo 2016	A	49.3	105	OW	KSS knee score KSS function score Lysholm	61.2 59.3 63.6	86.6 ± 5.9* 87.2 ± 7.2* 95 ± 7.6*

FU: follow up; OA: medial osteoarthritis; KSS: Knee Society score; HSS: Hospital for Special Surgery score; WOMAC: Western Ontario McMaster Universities Osteoarthritis Index; VAS: Visual analogue scale for pain; OW: opening wedge; CW: closing wedge; FU: NR.: not reported; -1: patient series 1; -2: patient series 2; A: advanced OA (Kellgren-Lawrence ≥ 3, Ahlbach ≥ 2, or clearly stated advanced OA); VA: very advanced OA (K-L = 4 or Ahlbach ≥ 3); ˆ: significant difference compared with patient series-2/lower degree OA patients of the same study; *: statistically significant improvement compared with pre-operative value; N.R.: not reported

#### Knee society score (KSS)

The KSS was assessed in five studies [7, 26, 32, 38, 39]. Four out of five reported a range between 86.6 and 95.1 and between 81.5 and 95 at mid term follow-up for the knee and function sub-score, respectively. In all these studies the provided scores and changed were above the target values of PASS and MCID [18, 19]. One study reported data at long term follow up showing a range between 55.8 and 59.5 for the function sub-score in the very advanced OA and advanced OA group respectively [32]. The latter study represented the only one providing scores which were under the PASS target value.

#### Hospital for special surgery score (HSS)

The HSS was assessed in four studies [7, 26, 35, 37]. A score of 85.1, between 89.4 and 90.3, and 83 were reported for the HSS score at short-, mid- and long-term follow-up, respectively. All the reported changes between follow-up and preoperative evaluation were above the MCID [17].

#### Further scores

Two studies assessed the Oxford Knee Score (OKS) at short term follow up, reporting values between 42.8 ± 4.5 and 44, and between 39.2 ± 7.9 and 48 in the advanced and very advanced medial OA series, respectively [28, 29]. Both these studies presented scores higher than PASS [18]. In one of them [29] the pre-operative assessment was provided, revealing a significant improvement at two years follow up compared to baseline evaluation, with a change higher than the MCID [16]. The Western Ontario and McMaster Universities Osteoarthritis index (WOMAC) score was reported in two studies, ranging between 16.5 and 17.4 [35, 36] at a follow-up included between 24 and 40 months. The reported values were lower than the PASS score [20]. In the study in which pre-operative assessment was provided, the improvement at follow up was statistically significant [35], with an improvement higher than MCID [20]. The Lysholm score was assessed in two studies. Values between 87.4 and 95, both above the PASS, were reported, with a follow-up range between 40 and 105 months. The two studies provided pre-operative assessment, showing a statistically significant improvement in both of them [35, 39], with improvements higher than MCID. VAS scale was assessed in two studies, reporting values between 2.2 and 3.2 at final follow-up [35, 36]. In both these studies the provided VAS at follow up was lower then the PASS cut off values reported in literature [21]. KOOS was assessed in one study which reported significantly higher values for pain, symptoms and activity daily living KOOS sub-items at two years of follow up in patients with lower degree of OA compared to advanced OA group [27]. The subjective IKDC score was reported in one study, which showed a significant improvement from a pre-operative value of 44 to 65 at ten years of follow up [4]. One study reported the clinical outcomes as satisfaction rate, showing a percentage of satisfied patients of 65.7% at five-years follow-up [24].

## Discussion

The main finding of the current systematic review is that HTO in patients with advanced radiological medial knee OA showed a mean survivorship rate of 96.3% (range between 96.1% and 99.9%) and of 74.6% (range between 60% and 98.1%) at five and ten years of follow-up, respectively. Furthermore, good results in PROMs at short-, mid- and long-term follow up were reported in patients with a high degree of OA who underwent HTO, including significant functional improvement and pain relief in studies which reported pre-operative values. Thus, both the first and the second hypothesis of the study were validated.

These findings definitely challenge the dogma of not performing high tibial osteotomy in advanced osteoarthritis and confirm the well-established clinical track record of HTO procedure even in patients with an advanced degenerative status of the medial compartment at x-ray assessment.

The degree of OA has traditionally been considered a significant factor in choosing between a joint-preserving and a joint-replacing procedure, and the recommendation to perform osteotomies only in early-to-moderate OA was previously reported in the literature [3, 40]. Certainly, advanced medial OA may be responsible for making HTO procedure more challenging, due to several factors such as decrease in joint space and increase in joint line convergence angle. Furthermore, clear evidence about the impact of HTO on medial joint space width, and its relationship with the amount of bone correction and the management of soft tissues still lack [41]. Therefore, the number of younger patients with advanced OA undergoing knee replacement surgery is increasing [8, 42]. Nevertheless, reports revealed that younger age is a risk factor for higher septic and aseptic revision rates after primary and revision total knee arthroplasty [42]. A population-based cohort study including 54,276 patients who underwent total knee replacement estimated a lifetime risk of revision of 35% for patients between 50 and 54 years old, with a mean time to revision surgery for patients aged 50–59 years of 4.55 years [9]. When it comes to UKA and its risk of conversion to TKA, data available in literature are controversial [43, 44]. A recent retrospective analysis drawn from the French National Hospitals Database accounting for 108.007 patient, showed better mid term survival of HTO, despite the fact that its indication has decreased in favour of UKA [44]. High survivorship of UKA has been reported in the latest National Joint Replacement of England and Wales, accounting for a figure between 85% and 93% at 15 years of follow up. However, the rate of failure at 15 years of follow up rise up to about 20% among both male and female patients under 55 years old. Furthermore [45], UKA and TKA in younger patients can be disastrous when complications such as component loosing, failure and infection occur [35].

Survivorship rates reported in the current review showed that HTO represents a reliable solution to delay joint replacement in younger patients with advanced medial OA. Moreover, data about HTO survivorship from total knee arthroplasty over the time indicated in the current study, were similar to those reported in studies including various degrees of medial OA. A previous systematic review including studies investigating the overall survivorship of HTO for medial compartment OA revealed a survival rate between 86% and 100%, 64% and 97.6, and 44% and 93.2% at 5, 10 and 15 years of follow up, respectively [46]. Furthermore, the latter review found five studies reporting survivorship at 20 years follow up, showing a range between 46 and 85.1% [46]. The only study included in the current review investigating 20-years follow-up survivorship provided results similar to those located in the highest part of that range, with a survivorship percentage of 75.9% [31]. The alteration of native anatomy and mechanical axis, and existing implants may be responsible for increasing the complexity of primary TKA procedure after HTO [47]. Nevertheless, excellent long term survivorship from aseptic loosening and revision surgery were reported in literature among patients who underwent TKA after HTO [11, 48, 49]. These results indicated that performing HTO in patients with advanced OA would not affect the outcomes in case of subsequent conversion to joint replacement procedure. On the other hand, revision surgery of UKA represents a technically demanding procedure whose outcomes compared with primary TKA are still controversial [43, 50, 51].

In order to select the most suitable patients for HTO, analysis of predictive factors for HTO success or failure have been previously carried out. A recent multicentric study on 481 patients pointed out that the presence of a complete joint line narrowing negatively impacted HTO survivorship from TKA, and included an Ahlbäck grade greater or equal to 3 in a predictive score for HTO failure [52]. Certainly, the findings of the current study confirm those results, with two of the included series reporting a significantly higher risks of conversion to TKA at long term follow up in “very advanced OA” sub-group patients compared to the “advanced OA” [32, 33]. However, the satisfactory rate of survivorship provided by all the studies included in the current review, clearly supports the use of HTO even in patients with a complete preoperative joint line narrowing, with the aim to delay the need for TKA. When it comes to PROMs analysis, there are several concerns to be taken into account. The main clinical aims of HTO are relief of medial knee pain and improved function. While there is a consensus in the literature about significant improvement of clinical outcomes in young patients with early-to-moderate OA and high preoperative function scores [53], the results of HTO in patients with advanced OA are still debated [40]. Furthermore, improvements reported in outcomes of joint replacement procedures meant that results from HTO in end-staged OA should be closely analysed. A systematic review assessing functional outcomes after TKA in patients under 55-years-old indicated a knee KSS of 89.7 and a functional KSS of 81.1 at minimum five years of follow up [8]. Those data are very similar to the results provided in the current review at four-to-nine-years of follow-up, ranging between 86.6 and 95.1, and between 81.5 and 95 for the knee and function KSS, respectively. Results of the current review revealed that most of the included studies reporting PROMs improvements and absolute values provided results above MCID and PASS target scores indicated in literature, at short, mid and long term of follow-up [15–21]. Only one study reported an absolute KSS function score lower than the PSS [32] at long term follow up, with a statistically significant difference between K-L 4 group and K-L 3 and 2 patients, pointing out that end stage OA may be responsible for a decline of the clinical outcomes over time.

Furthermore, the findings of the current review revealed that the results in patients with advanced OA are comparable to those of the early-to-moderate OA reported in the literature [46, 54], with excellent scores at short- and mid- term follow up and satisfactory values maintained at long term follow up. A meta-analysis including 2582 cases comprehensive of all degrees of OA reported significant improvement in HSS, Lysholm and VAS score similar to those showed in the current systematic review [54]. In the studies included in current review providing long-term follow-up analysis, PROMs scores slightly decreased compared with shorter follow-up, while maintaining satisfactory values.

Regarding the analysis of the “very advanced OA” sub-group, there are some interesting data to be considered. Three out of four included studies performing a separate analysis in the “very advanced OA” subgroup in which survivorship data were reported, showed that K-L 4 or Ahlbäch 3 medial OA significantly affected the failure rate with respect to K-L 3 or Ahlbäch 2 patient series [30, 32, 33]. This data point out that end stage OA may increase the risk of conversion to total knee replacement after HTO compared with lower OA degrees. However, the survivorship at ten years follow up in the four “very advanced OA” series were similar with the survivorship data of joint replacement procedures in younger patients reported in the literature, ranging between 60% and 86%. Concerning the PROMs assessment, two out of three studies comparing patient-reported scores between “very advanced OA” status and advanced OA only status, reported significant better scores in the lower OA grade patients [28, 32, 36]. Nevertheless, values of scores assessed at short- and mid-term follow-up were very good in two out of three of these studies, compared with the target PASS reported in the literature. These findings suggested that HTO represents a worthwhile alternative treatment to metal resurfacing that must be always considered in treatment of relatively young patients affected by end-stage medial knee osteoarthritis.

Overall, the current review showed that HTO resulted in good patient reported outcomes in patients with advanced OA, delaying the need for joint replacement and avoiding the high failure risk associated with knee replacement in young patients [10]. Even though HTO has been around for a very long time and its implications were based off a previously set age old dogma, it is a sound treatment option for severe and very severe knee osteoarthritis.

It is acknowledged that this systematic review presents several limitations. First, most of the included studies were low level of evidence studies. Nevertheless, as far as authors knowledge, this represents the current body of the literature available on the subject matter. Further prospective studies with a randomised controlled design are needed to achieve a better knowledge about HTO in various OA status. Furthermore, studies performed from 1990 to 2021 were included, with significant differences in study population and surgical technique among them. Due to the heterogeneity of study design, assessed outcomes, and data provided, only a qualitative analysis was performed. Furthermore, in the current review only medial compartment OA was considered. OA degree of the other knee compartments were not considered, even though they may be related to clinical outcomes after HTO.

## Conclusion

Valgus-producing HTO in patients with advanced to end-staged medial OA presented good survival rate at mid and long-term follow up and satisfactory patient reported clinical outcomes. HTO is definitely worth considering as treatment for young-to-mid aged patients with advanced medial knee osteoarthritis.

## Abbreviations

HTO: high tibial osteotomy
OA: osteoarthritis
PROMs: patient-reported outcomes
PRISMA: Preferred Reporting Items for Systematic reviews and Meta-Analysis
K-L: Kellgren Lawrence
UKA: unicompartmental knee arthroplasty
TKA: total knee arthroplasty
MCID: minimal clinically important difference
PASS: patient acceptable symptom state
